# A Novel Multimodal Deep Image Analysis Model for Predicting Extraction/Non‐Extraction Decision

**DOI:** 10.1111/ocr.70057

**Published:** 2025-11-06

**Authors:** Sunna Imtiaz Ahmad, Jakub Olczyk, Adriel S. Araújo, João Pedro de Moura Medeiros, Vinicius C. Teixeira, Carlos F. A. Gomes, Maurício Cecílio Magnaguagno, Quinn Roederer, Vinicius Dutra, R. Scott Conley, Dalvan Griebler, George Eckert, Márcio Sarroglia Pinho, Hakan Turkkahraman

**Affiliations:** ^1^ School of Dentistry Indiana University Indianapolis Indiana USA; ^2^ School of Technology Pontifical Catholic University of Rio Grande do Sul Porto Alegre Brazil

**Keywords:** artificial intelligence, clinical decision‐making, deep learning, orthodontics, tooth extraction

## Abstract

**Objective:**

This study aimed to develop a deep learning model classifier capable of predicting the extraction/non‐extraction binary decision using lateral cephalometric radiographs (LCRs) and intraoral scans (IOS) to serve as an additional decision‐support tool for orthodontists.

**Materials and Methods:**

The dataset was composed of LCRs and IOS from 617 patients (mean age: 18.2, 63.5% female) treated at the Indiana University School of Dentistry. Subjects were categorised into two groups: extraction (192) and non‐extraction (425). Two sets of features were extracted from IOS: traditional arch measurements and novel tooth spatial features. For LCRs, features were derived using CephNet‐based landmark detection (Land), a convolutional autoencoder (AE), and the dimensionality was reduced using Principal Component Analysis (PCA). Models were evaluated using accuracy, sensitivity, specificity, positive predictive value (PPV or precision), negative predictive value (NPV), positive likelihood ratio (LR^+^), negative likelihood ratio (LR^−^), and F1 score.

**Results:**

IOS + Land model achieved the highest overall accuracy (77%) and F1 score (0.62), with strong specificity (83%) and PPV (62%). In contrast, the Land model yielded the highest sensitivity (82%), but at the cost of lower specificity (57%). McNemar's test revealed that the AE model was significantly less accurate than IOS + AE (*p* = 0.048), IOS + Land (*p* = 0.006), and IOS + AE + Land (*p* = 0.005).

**Conclusion:**

Deep learning models can predict the extraction/non‐extraction decision using IOS and LCRs with high accuracy and diagnostic performance. Multimodal approaches, particularly those integrating IOS with cephalometric landmarks, demonstrate superior accuracy, sensitivity, and specificity compared to single‐modality models.

## 1 Introduction

Malocclusion is a widespread dental condition that affects millions of people globally [1]. Orthodontic treatment plays an important role in correcting malocclusion, requiring careful planning and critical decision‐making to achieve optimal aesthetic and functional outcomes. One of the most significant, and historically contentious, decisions in the orthodontic treatment planning process is whether to extract teeth or not. This debate dates back to the early 1900s, when Edward H. Angle advocated that proper alignment could be achieved without removing any teeth. In contrast, one of his protégés, Charles Tweed, later argued for the selective use of extractions to enhance facial harmony and long‐term stability [2]. Despite decades of clinical advancements, the extraction versus non‐extraction debate still persists, driven by differences in clinical training, experience, and individual treatment philosophies.

Given the irreversible nature of removing teeth, this decision carries significant clinical implications. An inappropriate diagnosis or treatment plan can result in extended treatment times, unnecessary financial burdens, and compromised aesthetic or functional outcomes [3]. To address these challenges, artificial intelligence (AI)‐based clinical decision support systems (CDSS) have emerged as promising tools to aid orthodontists in making more informed, patient‐specific decisions [4]. Numerous studies have explored the use of AI and machine learning (ML) to improve orthodontic treatment planning, including determining the need for orthognathic surgery [5, 6, 7]. Furthermore, AI has demonstrated effectiveness as a predictive tool for the binary decision of extraction versus non‐extraction, with studies reporting prediction accuracies ranging from 79% to 92% [8, 9, 10, 11, 12, 13, 14, 15]. These supervised ML models incorporated quantitative and categorical cephalometric data, offering promising predictive capabilities. However, their reliance on manual data collection can result in increased method errors and restricted sample sizes.

Deep convolutional neural networks (DCNNs) offer a significant advantage by bypassing the need for manual landmark identification altogether, enabling them to capture complex features often missed by traditional landmark‐based methods. Previous studies developed a DCNN‐based AI model that achieved over 89% accuracy in classifying sagittal and vertical skeletal relationships directly from lateral cephalometric radiographs (LCRs) without manually landmarking the cephalometric variables [16, 17, 18]. Expanding on this, Nan et al. incorporated multimodal inputs by combining LCRs with profile photographs and achieved an accuracy of 91.4% in skeletal classification [19]. Lee et al. advanced the application of DCNNs further by designing models to use LCRs to differentiate indications for orthognathic surgery, achieving an accuracy of up to 96.4% [20]. Similarly, Ryu et al. applied deep learning to intraoral photographs, achieving high performance in both dental crowding categorisation and extraction decision‐making, with their VGG19 model reaching an extraction prediction accuracy of 92.2% [21]. While these studies demonstrate the potential for image‐based skeletal and treatment classification tasks, a gap remains in targeting the binary extraction versus non‐extraction decision specifically using multimodal imaging inputs. Preliminary work focused on developing a deep image‐based ML model that utilises 3D intraoral scans (IOS) to predict the orthodontic extraction versus non‐extraction decision [22, 23]. By leveraging deep learning for automated tooth segmentation and engineering both traditional and novel dentoalveolar features, an ensemble of ML classifiers was trained, achieving a 73% accuracy in predicting extraction versus non‐extraction cases [23]. These early results highlight the potential of IOS‐based image analysis for clinical decision support and provide a strong foundation for expanded model development.

The aim of this study is to take it a step further and develop an image‐based ML classifier that integrates information from both LCRs and IOS to help predict the decision of extraction versus non‐extraction in orthodontic treatment planning. This classifier is intended to serve as an additional decision‐support tool for orthodontists, offering valuable insights to enhance treatment planning. To the best of current knowledge, no study to date has utilised AI‐driven image analysis of IOS and LCRs to support clinical decision‐making regarding tooth extractions.

The specific aims of this study were to (1) develop a deep learning model using IOS and LCRs to predict extraction versus non‐extraction decision and (2) test the model's performance by evaluating predicted outcomes with actual outcomes. We hypothesize that we will be able to create a novel predictive model of extraction/non‐extraction decision that can perform at comparable accuracy to that of a clinical orthodontist.

## 2 Materials and Methods

### 2.1 Ethics

This study was approved by the Institutional Review Board of Indiana University (Protocol #: 24369 August 29th, 2024). This study was deemed exempt, under Category 4 (iii), with a waiver of authorisation granted in accordance with 45 CFR 164.512 (i). Given the use of de‐identified retrospective data, the requirement for informed consent was waived.

### 2.2 Study Sample

The data for this retrospective study consisted of 617 patients (mean age ± SD: 18.2 ± 7.9 years, 63.5% female) who were treated at the Indiana University School of Dentistry (IUSD) Graduate Orthodontic Clinic from 2010 to the present day. Inclusion criteria consisted of patients who: (1) were at least 13 years of age; (2) presented with permanent dentition present up to first molars; (3) included pre‐treatment IOS with bite registration and LCR. Patients with craniofacial anomalies, systemic disease, missing teeth, and IOS or LCRs with artefacts or missing structures were excluded from this study. IOS were obtained using Carestream (Carestream Dental LLC, Atlanta, GA) and Itero scanners (Align Technology, San Jose, CA) and LCRs were obtained using Carestream model 9000 (Carestream Dental LLC, Atlanta, Georgia).

### 2.3 Data Collection

Patient charts were identified using procedure codes from the IUSD Electronic Health Record (EHR) system, Axium (version 2024.2.01.43897). Two investigators (SA, QR) reviewed each chart and identified patients that met the inclusion criteria. Pretreatment IOS and LCRs were then de‐identified and extracted from each patient chart and stored in a secure Microsoft Teams folder and shared via a secure share link by the university's IT department. The subjects were categorised into two exclusive treatment groups, extraction (*n* = 192, 31.1%) and non‐extraction (*n* = 425, 68.9%), according to the original treatment plan option created by a diverse set of experienced faculty members and residents.

### 2.4 Sample Allocation

The dataset was randomly partitioned into training and testing subsets. Stratification was performed based on the binary extraction outcome to preserve class proportions across both groups. Approximately 80% of the dataset (493 subjects) was allocated to the training set for model development and cross‐validation, while the remaining 20% (124 subjects) was reserved for the hold‐out test set for the final performance evaluation.

### 2.5 Data Preprocessing and Feature Extraction

**FIGURE 1:**
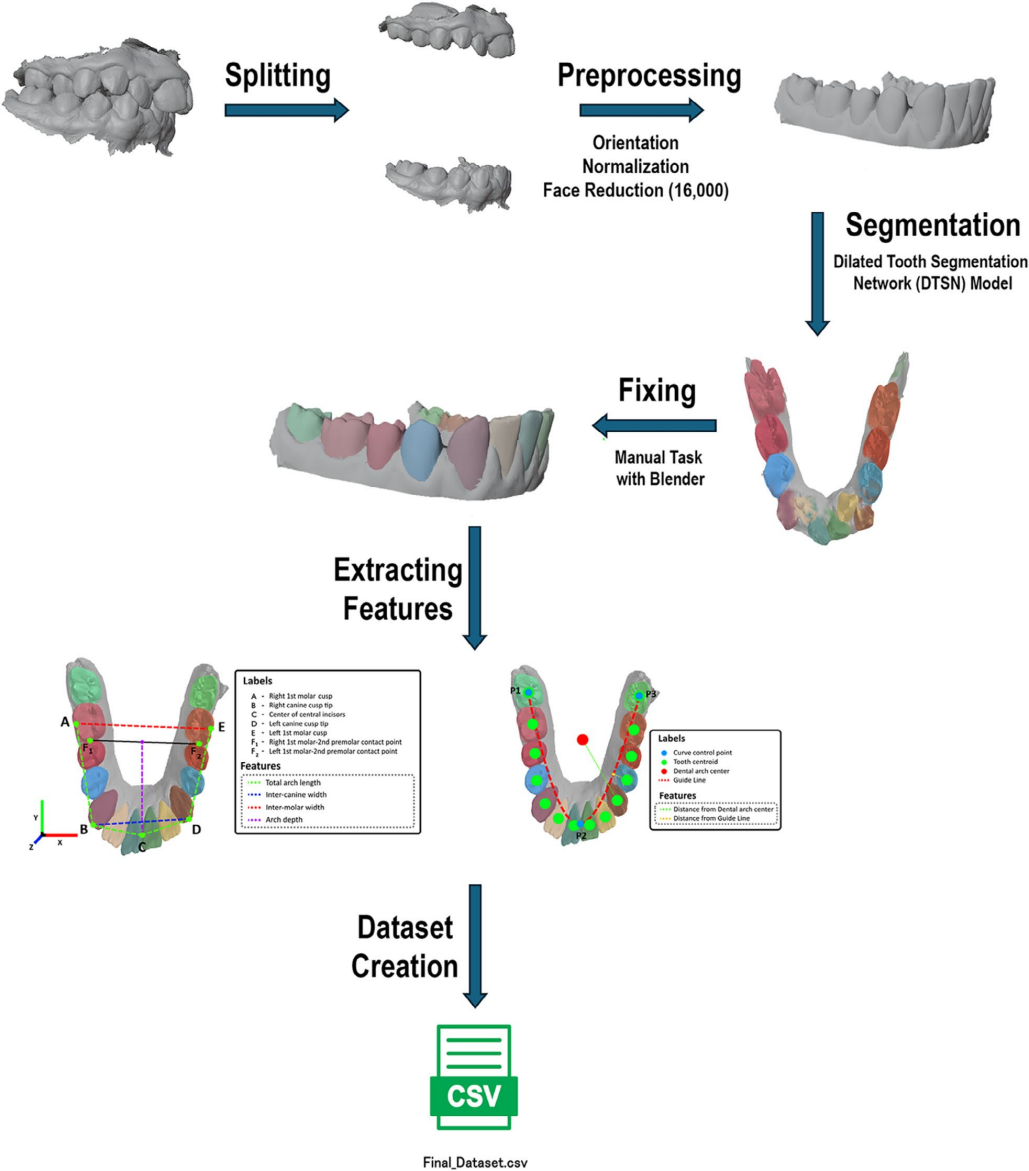
Preprocessing and feature extraction pipeline for the IOS data. IOS were divided into maxillary and mandibular arches, oriented, standardised, and simplified. Tooth segmentation was performed using the Dilated Tooth Segmentation Network (DTSN), followed by manual corrections with Blender. From the finalised segmentations, four standard orthodontic features (arch length, total inter‐canine width, total inter‐molar width, arch depth) and two novel geometric features were extracted to create the final dataset.

Preprocessing and feature extraction pipeline of the IOS data is shown in Figure 1. The procedure begins by dividing each 3D scan into separate files for the maxillary and mandibular arches. Each model was then standardised by defining a consistent orientation, normalising the *X*, *Y*, and *Z* axes, and reducing the mesh complexity to 16,000 faces. Afterward, individual teeth were segmented using the Dilated Tooth Segmentation Network (DTSN) [24], trained on 1800 labelled IOS from the Teeth3DS+ dataset [25]. The model achieved a test accuracy of 94.9% and a mean IoU of 0.838. This trained model was applied to segment 617 unlabeled IOS from our sample. Four non‐expert examiners manually corrected mislabeled faces using Blender (Blender Foundation, Amsterdam, Netherlands). From the segmented scans, four standard orthodontic features were extracted including total arch length, inter‐molar width, inter‐canine width, and arch depth. Additionally, two novel features were computed as previously described in detail: [23] (1) the distance from each tooth's centroid to the arch center and (2) the distance to a quadratic Bézier curve fitted through the 2nd molars and central incisors. These features provided geometric context and improved model performance.

**FIGURE 2:**
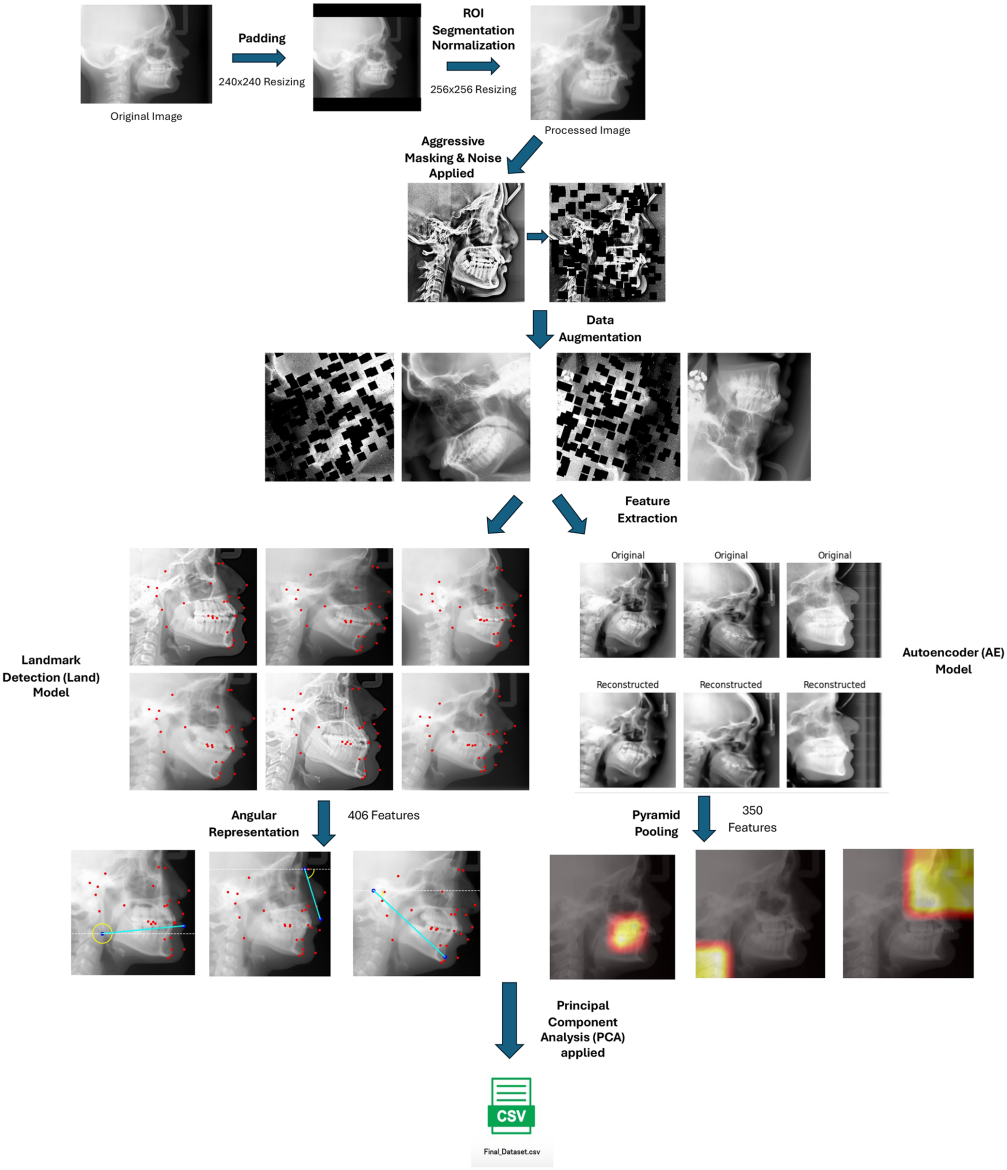
Preprocessing and feature extraction pipeline for the LCR data. Images were standardised through grayscale conversion, padding, resizing, and normalisation. Additional preprocessing steps were implemented, such as noise injection, masking, and data augmentation. Two deep learning models were used to extract features: a landmark‐based model (Land) generating 406 angular features and an autoencoder (AE) model with spatial attention and pyramid pooling producing 350 features. Principal Component Analysis (PCA) was applied to reduce feature dimensionality before creating the final dataset.

Preprocessing and feature extraction pipeline for the LCRs is illustrated in Figure 2. All input images were first converted to grayscale and processed with histogram equalisation. The images were then symmetrically padded with zeros to form square dimensions, preserving the original aspect ratio without distortion. Each padded image was resized to 240 × 240 pixels using bicubic interpolation and normalised to the range [0,1]. This was done so that a trained bounding box detector could be applied to identify a region of interest and exclude the background. The predicted bounding box coordinates were scaled back to the original image space, accounting for any padding added during squaring. The corresponding region was then cropped from the original grayscale image and resized to a fixed output resolution of 256 × 256 pixels using bicubic interpolation. To further enhance model robustness and generalisability, additional preprocessing was applied: noise was introduced to simulate real‐world variability; aggressive masking was performed by occluding portions of the image to encourage the model to learn meaningful spatial features; and a series of augmentation techniques, including random rotations, translations, and zooming, were implemented to replicate variations in head orientation and imaging angles typically observed in clinical settings.

For the LCRs, two types of deep learning models were used to extract features from the preprocessed images. The first model was based on traditional cephalometric landmarks (Land), and the second model on an autoencoder (AE) architecture. The Land model was based on a modified CephNet architecture [26] implemented to automatically detect 19 cephalometric landmarks using the Aariz and Cepha400 dataset [27, 28]. To capture the geometric configuration of anatomical landmarks in a compact and relational form, an angular feature representation was introduced, derived from the predicted landmark coordinates. Given a set of 2D landmarks produced by a neural network model, specifically, 29 paired (*x*, *y*) coordinates corresponding to cephalometric landmarks, the orientation angles between all unique point pairs were computed. This approach transforms the raw spatial output of the model into a geometry‐aware feature vector that is invariant to translation and less sensitive to scale and rotation. For each image, the predicted landmarks are arranged as ordered pairs (*x*_*i*_, *y*_*i*_) for *i* = 1, …, 29. The arc tangent of the line formed between each 2 points and the *x*‐axis was computed. This results in a total of 406 angular measurements per image, capturing the full pairwise relational geometry among the landmark set. The final output is a 406‐dimensional feature vector. By encoding only the relative orientation between landmarks, this representation serves as a generalised angular representation from the landmarks.

For the AE model, a fully convolutional AE architecture designed for grayscale image reconstruction was used, incorporating spatial attention and a spatially structured latent representation. The model eliminates fully connected layers, relying exclusively on convolutional and transposed convolutional operations to preserve spatial coherence throughout. Spatial attention modules are integrated after each convolutional block, enabling the network to emphasise informative regions via learned attention maps. The encoder compresses the input image through three convolutional stages with increasing filter sizes (32, 64, 128) and stride‐2 downsampling, producing a 32 × 32 × 128 feature map. A 1 × 1 convolution reduces this to a compact 32 × 32 × 4 spatial embedding, maintaining structural detail. The decoder symmetrically upsamples this embedding using transposed convolutions to reconstruct the original image dimensions.

To extract fixed‐length descriptors from the spatial embedding, a Spatial Pyramid Pooling (SPP) module is applied. Features were pooled across multiple spatial scales ([1 × 1], [2 × 2], [4 × 4], [8 × 8]), generating 85 distinct regions, each represented by four channels, resulting in a 340‐dimensional feature vector. This design ensures multi‐scale sensitivity and structural fidelity without early spatial flattening. Poorly cropped or mislabeled data were removed, resulting in a final dataset of 617 processed LCRs.

Principal Component Analysis (PCA) was applied to all LCR features to reduce dimensionality and prevent overfitting, especially given the feature‐to‐sample imbalance (756 features, 617 samples).

### 2.6 Training

To identify the most effective classifier for the extraction classification, a nested cross‐validation framework combined with Bayesian optimisation for model selection and hyperparameter tuning, using Python libraries' *scikit‐learn* for the models' training and *scikit‐optimise* for the optimisation [29, 30]. Four supervised learning algorithms were evaluated: logistic regression, support vector classifier (SVC), random forest, and decision tree. Each model was tuned over a relevant hyperparameter space using Gaussian Process‐based optimisation.

Feature vectors were derived from three groups, IOS, AE, and Land, and processed using *Z*‐score scaling and PCA, retaining 95% of variance. PCA was primarily applied to LCRs, due to the large number of features generated, thus attempting to reduce the model's susceptibility to overfitting. The resulting components were concatenated to form the final input space.

Model performance was evaluated via *F*1‐score in a stratified 5‐fold outer cross‐validation, with a 10‐fold inner loop for tuning for 100 tries. The best model from each outer fold was retrained and tested on its respective held‐out set. Performance across models was compared based on aggregated outer‐fold *F*1‐scores. The SVC emerged as the top‐performing model under this procedure, with the highest mean *F*1 score of 0.57, followed by logistic regression with a mean *F*1 score of 0.55. Random Forest and Decision Tree models had lower *F*1 scores of 0.50, with Random Forest showing more variability in performance.

**FIGURE 3:**
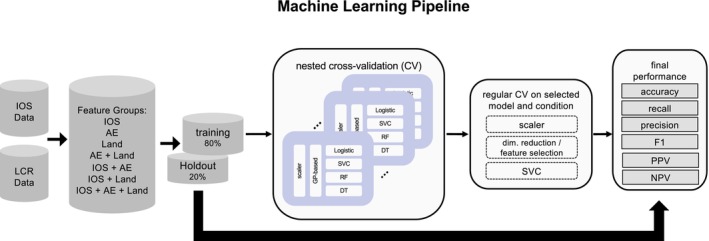
Model training and testing pipeline. A nested cross‐validation framework was used to train SVC models using Bayesian optimisation for hyperparameter tuning. Features derived from IOS, AE, and Land were evaluated in seven different configurations. SVC models were trained and evaluated using stratified 5‐fold outer and 10‐fold inner loops. Model performance was assessed using accuracy, recall, precision, F1, PPV, and NPV.

Following the comparison of model performance, hyperparameter optimisation was performed for the SVC models using three distinct feature groups: IOS, AE, and Land. A total of seven feature configurations were evaluated: IOS, AE, Land, AE + Land, IOS + AE, IOS + Land, and IOS + AE + Land, comprising individual modalities, pairwise combinations, and the full multimodal set. Figure 3 provides an overview of the nested cross‐validation framework, the seven evaluated feature configurations, and the performance metrics used for comparison. Each feature group was preprocessed independently with *Z*‐score standardisation and PCA. Preprocessing was conducted within folds to prevent data leakage, and all transformation components were saved per fold. For each configuration, a 5‐fold outer and 10‐fold inner nested cross‐validation was employed to tune SVC hyperparameters and assess generalisation performance using the *F*1‐score. Models were retrained on the full training fold using the optimal parameters and evaluated on the hold‐out set. All trained models, preprocessing pipelines, and performance metrics were stored for reproducibility. An ensemble prediction pipeline was also implemented, using majority voting across saved models to classify unseen data. This procedure yielded a ranked comparison of feature group combinations by *F*1‐score and a deployable ensemble of trained SV classifiers with corresponding preprocessing workflows.

### 2.7 Model Testing

Model performance was evaluated using accuracy (percentage of correctly assigned extraction/non‐extraction patients), sensitivity (also known as recall), specificity, positive predicted value (PPV, also known as precision), negative predicted value (NPV), positive likelihood ratio (LR^+^), negative likelihood ratio (LR^−^), and *F*1 scores. This integrated approach allowed the model to leverage both 3D intraoral geometry and 2D craniofacial structure, offering a comprehensive basis for extraction decision prediction.

### 2.8 Statistical Analyses

Models were compared for differences in accuracy, sensitivity, and specificity using McNemar's tests. A two‐sided 5% significance level was used for all tests. Analyses were performed using SAS version 9.4 (SAS Institute Inc., Cary, NC).

## 3 Results

The test dataset consisted of 124 cases, comprising 38 extraction cases (31%) and 86 non‐extraction cases (69%). Diagnostic performance metrics were evaluated across several models to assess their effectiveness in classifying extraction versus non‐extraction treatment plans, as shown in Table 1. The IOS + Land model achieved the highest accuracy (0.77), sensitivity (0.63), and positive predictive value (0.62), making it the most effective model overall. The AE model showed the lowest performance, with accuracy (0.61) and sensitivity (0.50) both relatively low. Combining IOS, AE, and Land produced moderate performance with an accuracy of 0.75 and sensitivity of 0.58. The Land and IOS + Land models also showed good specificity, but the models with only AE and AE + Land exhibited lower sensitivity and PPV.

**TABLE 1:** Performance metrics for each model in classifying orthodontic extraction versus non‐extraction cases.

Model	Accuracy	Sensitivity	Specificity	PPV	NPV	LR^+^	LR^−^	*F*1
IOS	0.68	0.68	0.67	0.48	0.83	2.10	0.47	0.57
AE	0.61	0.50	0.66	0.40	0.75	1.48	0.75	0.44
Land	0.65	0.82	0.57	0.46	0.88	1.90	0.32	0.58
AE + Land	0.69	0.39	0.83	0.50	0.76	2.26	0.73	0.44
IOS + AE	0.70	0.61	0.74	0.51	0.81	2.37	0.53	0.55
IOS + Land	0.77	0.63	0.83	0.62	0.84	3.62	0.45	0.62
IOS + AE + Land	0.75	0.58	0.83	0.59	0.82	3.32	0.51	0.59

*Note:* Metrics include overall accuracy (% correct), sensitivity (recall), specificity, positive predictive value (PPV or precision), negative predictive value (NPV), positive likelihood ratio (LR^+^), negative likelihood ratio (LR^−^), and *F*1 score. Accuracy calculations were based on treatment plans determined by the attending faculty and resident; patient preferences were not considered in defining the ground‐truth labels.

### 3.1 Comparison of Classification Accuracy Between Models

To evaluate differences in overall classification accuracy between models, pairwise comparisons were conducted using McNemar's test, as shown in Table 2. The AE model demonstrated significantly lower accuracy compared to IOS + AE (*p* = 0.048), IOS + Land (*p* = 0.006), and IOS + AE + Land (*p* = 0.005). No other pairwise comparisons yielded statistically significant differences.

**TABLE 2:** Pairwise comparisons of classification accuracy (% correct) across models using McNemar's test.

Comparison	Result	*p*	Significance
IOS versus AE	IOS & AE	0.258	
IOS versus Land	IOS & Land	0.599	
IOS versus IOS + AE	IOS & IOS + AE	0.612	
IOS versus IOS + Land	IOS & IOS + Land	0.056	
IOS versus AE + Land	IOS & AE + Land	0.768	
IOS versus IOS + AE + Land	IOS & IOS + AE + Land	0.117	
AE versus Land	AE & Land	0.593	
AE versus IOS + AE	AE < IOS + AE	0.048	*
AE versus IOS + Land	AE < IOS + Land	0.006	**
AE versus AE + Land	AE & AE + Land	0.096	
AE versus IOS + AE + Land	AE < IOS + AE + Land	0.005	**
Land versus IOS + AE	Land & IOS + AE	0.345	
Land versus IOS + Land	Land < IOS + Land	0.019	*
Land versus AE + Land	Land & AE + Land	0.396	
Land versus IOS + AE + Land	Land & IOS + AE + Land	0.053	
IOS + AE versus IOS + Land	IOS + AE & IOS + Land	0.131	
IOS + AE versus AE + Land	IOS + AE & AE + Land	0.862	
IOS + AE versus IOS + AE + Land	IOS + AE & IOS + AE + Land	0.221	
IOS + Land versus AE + Land	IOS + Land & AE + Land	0.139	
IOS + Land versus IOS + AE + Land	IOS + Land & IOS + AE + Land	0.637	
AE + Land versus IOS + AE + Land	AE + Land & IOS + AE + Land	0.144	

*Note:* Asterisks indicate statistically significant differences: *p ≤ 0.05; **p ≤ 0.01.

### 3.2 Pairwise Comparisons of Sensitivity and Specificity

To further evaluate model performance, sensitivity and specificity were compared across all model pairs using McNemar's test to determine whether any models showed significant differences in their ability to correctly identify extraction (sensitivity) and non‐extraction (specificity) cases, as shown in Table 3. The Land model shows significantly higher sensitivity than AE, AE + Land, and IOS + AE + Land models with *p*‐values of 0.005, < 0.001, 0.020 respectively. However, its specificity was significantly lower than IOS + AE, IOS + Land, AE + Land, IOS + AE + Land with *p*‐values of 0.019, < 0.001, < 0.001, and < 0.001, respectively.

**TABLE 3:** Pairwise comparisons of model performance for sensitivity and specificity.

Comparison	Sensitivity			Specificity		
	Result	*p*	Significance	Result	*p*	Significance
IOS versus AE	IOS & AE	0.052		IOS & AE	0.869	
IOS versus Land	IOS & Land	0.166		IOS & Land	0.180	
IOS versus IOS + AE	IOS & IOS + AE	0.366		IOS & IOS + AE	0.221	
IOS versus IOS + Land	IOS & IOS + Land	0.527		IOS < IOS + Land	0.007	**
IOS versus AE + Land	IOS > AE + Land	0.008	**	IOS < AE + Land	0.016	*
IOS versus IOS + AE + Land	IOS & IOS + AE + Land	0.206		IOS < IOS + AE + Land	0.007	***
AE versus Land	AE < Land	0.005	**	AE & Land	0.194	
AE versus IOS + AE	AE & IOS + AE	0.157		AE & IOS + AE	0.144	
AE versus IOS + Land	AE & IOS + Land	0.225		AE < IOS + Land	0.011	*
AE versus AE + Land	AE & AE + Land	0.206		AE < AE + Land	0.006	**
AE versus IOS + AE + Land	AE & IOS + AE + Land	0.366		AE < IOS + AE + Land	0.006	**
Land versus IOS + AE	Land > IOS + AE	0.033	*	Land < IOS + AE	0.019	*
Land versus IOS + Land	Land & IOS + Land	0.052		Land < IOS + Land	< 0.001	***
Land versus AE + Land	Land > AE + Land	< 0.001	***	Land < AE + Land	< 0.001	***
Land versus IOS + AE + Land	Land > IOS + AE + Land	0.020	*	Land < IOS + AE + Land	< 0.001	***
IOS + AE versus IOS + Land	IOS + AE & IOS + Land	0.739		IOS + AE & IOS + Land	0.108	
IOS + AE versus AE + Land	IOS + AE > AE + Land	0.033	*	IOS + AE & AE + Land	0.108	
IOS + AE versus IOS + AE + Land	IOS + AE & IOS + AE + Land	0.739		IOS + AE & IOS + AE + Land	0.071	
IOS + Land versus AE + Land	IOS + Land > AE + Land	0.050	*	IOS + Land & AE + Land	1.000	
IOS + Land versus IOS + AE + Land	IOS + Land & IOS + AE + Land	0.527		IOS + Land & IOS + AE + Land	1.000	
AE + Land versus IOS + AE + Land	AE + Land < IOS + AE + Land	0.035	*	AE + Land & IOS + AE + Land	1.000	

*Note:* Results indicate whether one model outperformed another in detecting extraction (sensitivity) or non‐extraction (specificity) cases. Asterisks denote statistically significant differences: *p ≤ 0.05; **p ≤ 0.01; ***p ≤ 0.001.

## 4 Discussion

This study was designed to provide an objective support system for clinicians in orthodontic decision‐making. The ground‐truth labels for extraction versus non‐extraction decisions were based on treatment plans determined jointly by the attending faculty and the resident, representing the most clinically informed and objective decisions available for algorithmic training. We acknowledge, however, that consensus‐based ground‐truth determination by multiple experts would represent the most rigorous standard and would help minimise potential label noise. It is also important to note that the algorithm's predictions may not always align with the final treatment choice, as clinicians must also weigh subjective factors such as patient and parent preferences, including aesthetic concerns, treatment duration, cost, or fear of profile flattening. While highly relevant in individual cases, these influences cannot be generalised across a broad patient population and were not incorporated into the present model. In addition, the algorithm was not trained with data reflecting such preferences (e.g., socioeconomic status or other personal considerations). Therefore, the proposed model should be viewed as an adjunct to, rather than a replacement for, clinical judgement. Future research should aim to integrate patient‐reported outcomes and subjective preferences into predictive modelling to provide a more comprehensive and patient‐centered decision support system.

Previous studies on AI applications in orthodontic diagnosis have largely relied on manually identified features to support clinical decision‐making. However, such approaches are limited by their reliance on predefined measurements, which may introduce feature selection bias and oversimplify complex anatomical relationships [8, 9, 10, 11, 12, 13, 14, 15, 21, 31, 32]. Recent work has begun to explore image‐based methods, such as convolutional neural networks (CNNs) and AE, yet the majority still depend heavily on single‐modality data [16, 17, 18, 20, 21]. There is a growing need for comparative studies assessing the benefits of multimodal approaches that integrate different types of clinical information.

In this study, models that incorporated multiple input modalities, specifically combinations involving IOS and LCR features, consistently outperformed those relying on a single modality. The AE and Land models showed significantly lower performance than their multimodal counterparts, highlighting the benefit of combining cephalometric data with three‐dimensional surface information from IOS. Among the evaluated models, the IOS + Land configuration achieved the highest overall accuracy at 77%. Although the accuracy of the machine learning approaches reported in prior studies was greater (79%–92%), it aligns with the variability seen among clinical orthodontists in the literature [8, 9, 10, 11, 12, 13, 14, 15]. Previous studies have reported orthodontist agreement rates for various orthodontic treatment decisions ranging from 58% to 72% [33, 34, 35]. For example, Han et al. evaluated 57 Class II cases and found that treatment plans based on study models match the full diagnostic set in only 54.9% of the cases. This agreement improved by 6% with the addition of radiographs, similar to the performance gain observed with the use of multimodal data [35]. These findings suggest that deep learning models can achieve a diagnostic accuracy comparable to that of practicing orthodontists, reinforcing their potential as adjunct tools to support decision‐making.

Building on the classification accuracy results, the models' diagnostic capabilities were further examined by comparing their sensitivity and specificity. This analysis revealed a trade‐off between identifying true extraction cases and avoiding false positives. The Land model achieved the highest sensitivity among all models, indicating a strong performance in correctly identifying patients requiring extraction. However, this came at the cost of reduced specificity, leading to a higher rate of false positives, which is an important concern in the context of extraction decisions, where irreversible tooth removal must be avoided unless clearly justified. In contrast, multimodal models that combined IOS with either AE or Land (such as IOS + Land and IOS + AE + Land) demonstrated higher specificity than the IOS model, suggesting an improved ability to correctly identify extraction cases. Although prior studies have questioned the clinical utility of LCRs in treatment planning, our findings suggest that when integrated into deep learning frameworks, LCR data provided valuable skeletal context that improved the model's specificity and overall predictive performance [36, 37, 38, 39].

While these findings highlight the potential of image‐based models to enhance diagnostic precision in orthodontic treatment planning, several limitations should be considered when interpreting the results. First, we analysed a dataset of 617 subjects, which is relatively large compared with previous studies in this domain. Nevertheless, we acknowledge that this number is still far from the limits of so‐called ‘big data.’ For this reason, the present work should be regarded as a proof‐of‐concept study that demonstrates the feasibility and potential of applying multimodal AI approaches to orthodontic decision‐making processes. Future studies incorporating larger and more diverse datasets will be essential to further validate and generalise our findings. Second, the interpretability of the final model is limited due to the ‘black box’ nature of neural networks and the dimensionality reduction applied through PCA, which obscured the influence of individual features on the predictions. This challenge could be addressed in future work through the use of interpretability tools like Class Activation Mapping (CAM) and optimising the number of features and increasing the sample size to avoid having to apply PCA [16, 20]. Additionally, the class imbalance between the extraction and non‐extraction cases, though reflective of real‐world clinical distributions, may have impacted the model's ability to accurately learn minority class patterns [40] To address this, oversampling and data augmentation techniques were implemented; however, future efforts may benefit from including more extraction cases and exploring other rebalancing strategies to further improve the model's performance. The external validity of our model is another consideration. Because the dataset was derived from a university clinic, where more severe malocclusions are commonly treated and extraction decisions may be more frequent, the algorithm may over‐recommend extractions if applied in private practice settings. In fee‐for‐service environments, patients often present with borderline cases and may prefer non‐extraction treatment options to minimise aesthetic changes such as profile flattening. As such, the present model may reflect the treatment biases of a university‐based population rather than the broader orthodontic community. To improve generalisability, future studies should incorporate data from diverse practice settings, including private clinics, to balance the representation of extraction and non‐extraction cases. Finally, patient‐reported satisfaction and psychosocial impact were not integrated into the ground‐truth label; therefore, the ‘ground truth’ decision remains clinician‐centered rather than patient‐centered.

## 5 Conclusions

- A deep learning model trained exclusively on intraoral scans achieved strong performance in predicting extraction versus non‐extraction decision.
- Models trained solely on cephalometric data achieved a modest accuracy, but further improvement is needed for better applicability.
- Deep learning models using multimodal inputs, especially combinations of intraoral scans with cephalometric landmarks or autoencoder‐derived features, achieved better accuracy than single‐modality models.

## Conflicts of Interest

The authors declare no conflicts of interest.

## Data Availability

The data that support the findings of this study are available on request from the corresponding author. The data are not publicly available due to privacy or ethical restrictions.
